# Addressing Barriers and Facilitators to African Americans’ and Hispanics’ Participation in Clinical and Genomic Research Through a Bioethical Sensitive Video

**DOI:** 10.1007/s13187-024-02433-w

**Published:** 2024-05-02

**Authors:** Victoria Churchill, Yu-Mei Schoenberger, Vivian L. Carter, Jamirah Y. Chevrin, Windy Dean-Colomb, Roland Matthews, Desiree Rivers, Stephen O. Sodeke, Jonathan Ezer, Brian M. Rivers

**Affiliations:** 1 Cancer Health Equity Institute, Morehouse School of Medicine, 720 Westview Dr SW, Atlanta, GA 30310 USA; 2 O’Neal Comprehensive Cancer Center, University of Alabama at Birmingham, 1824 6th Ave S, Birmingham, AL 35233 USA; 3 Center for Biomedical Research, Tuskegee University, 1200 W Montgomery Rd, Tuskegee, AL 36088 USA; 4 Piedmont Cancer Institute, 775 Poplar Rd, Suite 310 Newnan, Newnan, GA 30265 USA; 5 Kindea Labs, 70 Little W St #31E, New York, NY 10004 USA

**Keywords:** Cancer health disparities, Communication, Clinical research, Genomic education

## Abstract

Research advances on effective methods to prevent, diagnose, and treat cancer continue to emerge through clinical and genomic research. Most clinical trial and genomic research participants identify as White which limits the generalizability of research findings to non-White populations. With the development and access to technology, digital delivery of salient and tailored health education may provide innovative pathways to increase representation of African Americans (AA) and Hispanics in research. This project focused on the creation of a bioethical sensitive education video aimed at increasing participation in clinical trials and genomic research by bringing together experts from the community, healthcare, biomedical research, and public health. The goal was to utilize existing educational resources to create a tailored message to address AA/Hispanics’ beliefs, values, and bioethical concerns related to participation in clinical and genomic research. Models of behavior change and communication theories were leveraged to frame key components of the message, which then informed the framework for the animated video. Development of the video consisted of six iterative phases: 1) writing sessions; 2) storyboarding; 3) animating; 4) screening/revisions; 5) acceptability testing; 6) finalization. The final animated video is approximately 5 min in length and covers several topics including the goal of clinical research, disparities in research participation, bioethical concerns, and genomic research regulations. Increasing AA and Hispanic participation in clinical and genomic research is imperative to achieving health equity. Tailored messages via short videos may assist in addressing the barriers and facilitators towards research participation and increase intentions to enroll in trials.

**Supplementary Information:**

The online version contains supplementary material available at 10.1007/s13187-024-02433-w.

## Introduction

In the United States (US), nearly 17 million individuals live with a cancer diagnosis, and these numbers are projected to increase in the next 10 years as the population ages [1]. The quality of cancer care can affect survival and quality of life during and after cancer treatment. State-of-the-art cancer treatments are neither equitably accessible nor available across all segments of the population. As a result, disparities in cancer treatment and outcomes persist for medically underserved populations, such as racial/ethnic minority groups [2], persons who are uninsured or underinsured [3], and rural patients.

Advances in genomics have increased our understanding of the interplay between genetics, environment, behavior, and disease, which has led to the advancement of clinical trial-matching, precision oncology medicine, and personalized cancer prevention and treatment. However, these scientific advances are hampered by the inadequate representation of African American (AA) and Hispanic populations in biomedical research studies and clinical trials [4]. Clinical trials are research studies designed to evaluate the safety and efficacy of human interventions. To ensure that trial findings are generalizable to the public, participants should represent the patients who will be utilizing these products. However, racial/ethnic minority groups are underrepresented in most clinical trials, representing 5–11% of all trial participants [5]. In general, individuals often respond differently to medical products, and there is evidence that this is particularly relevant when taking into account race and ethnicity [6]. To address this issue the National Institutes of Health (NIH) Revitalization Act of 1993 directed the NIH to establish guidelines for the inclusion of women and minorities in clinical research. More recently, the Food and Drug Administration (FDA) issued recommendations to sponsors developing medical products on the approach for developing a Race and Ethnicity Diversity Plan to ensure adequate enrollment of participants in clinical trials from underrepresented racial and ethnic populations in the US [7]. However, the impact of those efforts has been hard to gauge, with limited progress noted [8].

Recent data from clinical trials and genome-wide association studies demonstrate the lack of representation of racial/ethnic minorities in biomedical research. The over-representation of European ancestry in these studies has been linked to reduced effectiveness of variant interpretation for individuals of non-European ancestry [9]. Implications related to this lack of representation include 1) an evidence base that does not reflect populations for whom the cancer burden may be greatest, and 2) enhanced potential for misdiagnosis that can widen healthcare disparities [10]. There is robust evidence describing a multi-factorial model that describes the lack of representation among racial/ethnic minority populations in research [11–13]. The most commonly cited factors to participation of racial/ethnic minority patients in clinical trials include systemic factors, such as strict exclusion criteria, cost, transportation, and convenience; patient-level factors including lack of awareness/knowledge of clinical trials available, misperceptions, mistrust of the health system, family pressures, religious beliefs, poor patient-provider communication, and concerns of privacy, trust, and safety [11–13].

While new developments in research on more effective methods to treat cancer continue to emerge through genomic research studies and clinical trials, it is imperative that individuals are provided with education on the importance of participation. However, there remains a paucity of culturally relevant genomic and clinical trial informational resources available to AA and Hispanic populations. Although health information resources regarding research participation exist online and in print, these methods may be outdated and not inclusive of the unique bioethical, social, and informational concerns of AA and Hispanic populations.

Digital delivery of research education may provide innovative pathways to target AA and Hispanic populations. Video interventions have been shown to be an effective method of disseminating health information to diverse groups, in part by delineating the impact of barriers to understanding health information, such as health literacy [14, 15]. However, their use for increasing clinical trial and genomic research participation, especially among racial/ethnic minority populations, has been mixed. One study among AA breast cancer patients who viewed a 15-min culturally tailored video on clinical trials demonstrated a significant increase in intention to enroll in a clinical trial, compared to baseline [16]. It is important to note, as the authors listed as limitations, that all participants in the study identified as female and it was a non-randomized design. In another study, cancer patients were randomly assigned to view a non-tailored educational video about clinical trial participation. At the end of the 1 year follow-up there was no statistically significant difference in clinical trial enrollment between the intervention and standard of care groups [17]. There is a need for tailored messages that address the negative perceptions of clinical trial participation, particularly among racial/ethnic minority populations.

This paper details the methods of developing a culturally tailored educational video aimed at increasing intentions to participate in genomic research and clinical trials among AA and Hispanic populations. We will discuss the steps from conception to initial delivery of the video to the target audiences. Future manuscripts will present findings from the randomized control trial comparing the existing modality of information delivery (i.e., National Cancer Institute [NCI] pamphlets) with the novel strategy that we present below.

## Methods

### Team Formation

A collaboration existed between Morehouse School of Medicine (MSM), Tuskegee University (TU), and University of Alabama at Birmingham (UAB), all of which are in the Southeast US, an area commonly referred to as the Deep South or the Black Belt Region and where the US Public Health Service Syphilis Study at Tuskegee was conducted. Considering the aim of the project—to increase AA and Hispanic participation in clinical trials, including genomic research and biobanking—it was imperative that experts across the spectrum of community, healthcare, and research were recruited to lend their expertise. For content expertise, oncologists with experience working with AA and Hispanic patients from all sites were asked to be leaders on the project. Experts in health communication and behavior change theory were added to the team, as well as researchers with expertise in program evaluation. As predominant research apprehensions among racial/ethnic minority populations are ethical concerns [13], a Bioethicist with proficiency in biomedical research was added. Finally, community navigators (CN) with insights on engaging racial/ethnic minority populations from all three sites were invited to participate.

### Guiding Frameworks and Theories

The decision was made to utilize theories of health behavior change and health communication to inform the script for the video. A literature review was conducted to identify potential messaging strategies to create interventions for AA and Hispanic communities. Initially we explored message framing. Messages can be designed using a variety of frames, and some have shown to be more effective at encouraging behavior changes. For example, messages can be “gain-framed” to highlight the benefits of taking action, or “loss-framed” to emphasize the disadvantages of not taking action. A study looking at gain vs loss-framed messages targeting colorectal cancer screening among AAs found that participants were more receptive to the gain-framed messages [18]. We also considered impact, progress, and disparity framing [19]. In a study of a sexual health messaging intervention among AAs, the messages with the impact frame were viewed as most trustworthy and they increased the intention to learn more and to get tested [20].

The Health Belief Model (HBM) is one of the most frequently utilized models to explain and change individual’s health behaviors. The model is comprised of five variables: perceived susceptibility, perceived severity, perceived benefits, perceived barriers, and cues to action. It has served as the basis for creating and evaluating communication-based strategies across many topics [21].

The Extended Parallel Process Model (EPPM) is a fear appeals theory that is often used in communication-based health interventions [22, 23]. This theory posits that when an individual encounters a message, they first assess their susceptibility and the severity of the threat. They then assess their ability to respond to the threat (self-efficacy) and how likely their response will control the threat (response efficacy). There are three outcomes in the EPPM: danger control, fear control, and no response. Messages that balance threat and self-efficacy are more likely to result in danger control, resulting in actions taken by the individual to control the threat [24].

While drafting the script for the video, our team was deliberate about including key phrases that aligned with the HBM and EPPM. The team also took care to weigh statements that communicated severity and susceptibility with ones that promoted self-efficacy.

## Results

There were six iterative stages in the development of the video: 1) brainstorming/writing; 2) storyboarding; 3) animating; 4) screening/revisions; 5) acceptability testing; 6) finalization, all of which are detailed below.

### Brainstorming/Writing Sessions

The team convened both in-person and virtually for a brainstorming and script writing session in Fall, 2022. In preparation, materials were distributed including pamphlets from the NCI (e.g., “Your Genome & You,” “Taking Part in Cancer Treatment Research Studies,” “Providing Your Tissue for Research”) and a presentation highlighting theories of behavior change and health communication was delivered. The team discussed which key elements from the NCI booklets to include in the script, and how to present them in ways that were engaging and appropriate for the target audiences. Based on guidance from the partnering media consulting agency (Kindea Labs), the goal was to keep the script under 1000 words, so that the video would not surpass 5-min, the maximum amount of time determined to be acceptable for the video’s purpose.

Specific phrases were written into the script that aligned with the HBM and the framing guides (Table 1). For example, the team wanted to communicate to the participants that they were susceptible to diseases for which there are clinical trials designed to prevent or treat. The corresponding text for this element reads: “Every human is at risk for getting a disease such as cancer.” To demonstrate severity, the team included the following: “People who have cancer or other serious diseases may get very sick and die, even with treatment.” For cues to action, the team included phrases including: “If you are interested in participating, please talk to the health navigator.” The phrasing in the script aimed to balance both the threat of the topic (i.e., disparities in clinical research) and efficacy of the participants (i.e., increasing participation in research among AA and Hispanic populations). Further analyses will be conducted during the implementation phase to assess if the message components elicited the appropriate responses (e.g., threat management) that are necessary for behavior change.

**Table 1:** Concepts from script related to theoretical model concepts

Model Construct or Frame	Script component
Cues to action	“But to do so, they need your help.”
Perceived susceptibility	“Every human is at risk for getting a disease such as cancer.”
Perceived severity	“People who have cancer or other serious diseases may get very sick and die, even with treatment.”
Perceived susceptibility	“These risks can be affected by things in our life that we do, such as what we eat and where we work.”
Self-efficacy	“…and you can contribute to this important work by participating in a clinical trial.”
Cues to action	“But to make sure that treatments work for everyone, a diverse group of people need to participate in clinical trials.”
Disparity frame	“Right now, only a small number of people, about 10%, who identify as African Americans and Latino make up clinical trial participants.”
Disparity frame	“There is an over-representation of people who identify as White in research, which means that the results from studies may not help those who need it the most. This can lead to and widen health disparities, meaning African American and Latinx populations may not benefit from advances in science, as much as their White counterparts.”
Cues to action	“Even if you don’t have a disease such as cancer, there are still studies you can join.”
Impact frame	“We know that people often respond differently to medical products, and this may be especially true for people of different races and ethnicities.”
Perceived benefits	“Therefore, having a diverse group of participants can help propel treatments that help the communities who need it the most.”
Perceived benefits	“When you participate in research you are helping to push science forward, and you may also gain new understandings about your health.”
Perceived barriers	“And remember, participation is voluntary. It's your choice whether you provide your DNA or participate in research.”
Perceived barriers	“There are many rules put in place to protect you if you decide to participate in a clinical trial, even if you decide to provide genetic samples. For example, the federal Genetic Information Nondiscrimination Act (GINA) prevents most employment and insurance discrimination based on genetic information. Your safety is always put first and you can end your participation in research at any time.”
Perceived benefits	“Many people find comfort knowing that by donating samples, they will help researchers make discoveries that can advance medicine and improve lives of people in their community, now and in the future.”
Cues to action	“If you are interested in participating, please talk to the health navigator.”

Once the team finalized a first draft of the script, it was sent to an external agency, Kindea Labs, with expertise in taking complex information and synthesizing it into language that is appropriate for delivery via animation. Kindea Labs completed an initial edit of the script, focused primarily on changing sentences and structures that did not lend themselves well to communication via animations, such as superfluous dialogue. They aimed for terse sentences and a symmetrical storyline (i.e., starting and ending on the same scene, see Supplemental Fig. 1a & d). After several more rounds of editing, the script draft was finalized and ready for storyboarding.

### Storyboarding

Kindea Labs reviewed the final script and identified visual metaphors for each sentence. For example, when the voiceover dictates, “This can lead to and widen health disparities, meaning AA and Hispanic populations may not benefit from advances in science, as much as their White counterparts” an image of a balance visually representing a disparity between a group of White and non-White individuals is shown (Supplemental Fig. 1b). In the present example, a prominent character is shown reading about clinical trials on a computer and discussing questions with a doctor (Supplemental Fig. 1c). Kindea Labs then presented the storyboard to the team for feedback before starting the animation process.

### Animating

Kindea Labs has several animating crews worldwide to artistically represent the script based on the storyboard. Reference images to people and places that would be familiar to the target audience (i.e., oncology physicians, clinic spaces) were provided by the research team and were incorporated as much as possible into the animation (Supplemental Fig. 1a & d).

### Screening/Revisions

Once the first draft of the animated video was completed, it was sent to the research team for an initial viewing. The team discussed what worked well and suggested edits to improve the video. The team members were also able to view the video on their own and offer feedback as the acceptability phase was underway.

### Acceptability Testing

#### Quantitative Feedback

As part of the study’s commitment to keeping the end-user (i.e., underrepresented groups in clinical trials) as the driver of the video design, the team decided to utilize the Theoretical Framework of Acceptability (TFA) to assess initial feedback to the video 25. A questionnaire was adapted from the seven components of the TFA: “affective attitude, burden, ethicality, intervention coherence, opportunity costs, perceived effectiveness, and self-efficacy” (Table 2). We used a convenience sample, which included members of a patient advisory council at a local hospital (*n* = 6) and local community members (*n* = 31) to assess acceptability.

**Table 2:** Theoretical framework of acceptability (TFA) adaption to questionnaire and results from preliminary testing (*n* = 37)

Construct	Assessment	Mean (SD)
Affective Attitude	I enjoyed listening to the audio in the video.	4.2 (1.0)^a^
	I enjoyed the illustrations in the video.	4.3 (1.0)^a^
Burden	How much effort did it take to understand the topics discussed in the video?	1.5 (0.8)^b^
Ethicality	The video was inclusive of my beliefs and values.	4.2 (0.8)^a^
Perceived effectiveness	The video educated me on what a genome is.	4.2 (0.8)^a^
	The video educated me on the benefits of participating in a clinical trial.	4.6 (0.5)^a^
	The video educated me on the benefits of donating biospecimens.	4.1 (0.9)^a^
	The video educated me on the disadvantages of participating in a clinical trial.	3.4 (1.4)^a^
	The video educated me on the disadvantages of donating biospecimens.	3.1 (1.2)^a^
	The video educated me on how I can sign up to participate in a clinical trial.	3.7 (1.1)^a^
	The video educated me on how I can sign up to donate biospecimens.	3.7 (1.1)^a^
Intervention coherence	After watching the video, it is clear how donating biospecimens advances science to improve the health of people in my community.	4.5 (0.5)^a^
	After watching the video, it is clear how participating in a clinical trial advances science to improve the health of people in my community.	4.6 (0.5)^a^
	After watching the video, it is clear to me how my genome can affect my risk of developing cancer.	4.2 (0.8)^a^
	After watching the video, it is clear how I can sign up for donating biospecimens.	3.4 (1.2)^a^
	After watching the video, it is clear how I can sign up for participating in a clinical trial.	3.6 (1.2)^a^
Self-efficacy	After watching this video, I am confident that if offered to participate in a clinical trial I am now knowledgeable enough to make a well-informed decision about participation.	4.3 (0.7)^a^
	After watching the video, I am confident that if offered to donate biospecimen I am now knowledgeable enough to make a well-informed decision about participation.	4.2 (0.8)^a^
Opportunity Costs	Watching this video interfered with my other priorities.	1.7 (1.0)^a^
	I spent too much of my time watching this video.	1.4 (0.5)^a^
General Acceptability	How acceptable was this video to you?	4.4 (1.0)^c^
^a^On a Likert scale from 1 = strongly disagree to 5 = strongly agree ^b^On a Likert scale from 1 = no effort at all to 5 = a lot of effort ^c^On a Likert scale from 1 = not at all acceptable to 5 = completely acceptable		

Overall, the video was well received by the preliminary testers, with almost all rating the video as “completely acceptable” (25/37, 68%) or “acceptable” (9/37, 24%). The findings of the Acceptability study were utilized to reflect the target population values, preferences and beliefs in the digital education tool.

#### Qualitative Feedback

A convenience sample of community members in Georgia were invited to participate in a viewing of the video followed by a guided question and answer session. A community outreach coordinator either identified and contacted directly through an internal list of community members, or were invited to participate through word-of-mouth. Eighteen participants attended the inaugural focus group (Table 3). They were asked about their trust in clinical research and then were shown the video. Afterwards, a moderated conversation occurred using pre-chosen questions, such as “What are your initial feelings towards the video?” and “Did you understand what the video was trying to say?” Qualitative feedback included that video was useful in “breaking down the terminology” and that it was “straight to the point.” The participants also commented on how the video highlighted the current racial disparities in clinical trials.

**Table 3:** Demographics of focus group participants (*N* = 18)

	*N* (%)
Age (years): Mean (range)	61 (36 – 85)
Female sex	12 (66.7%)
Race	
Black	17 (94.4%)
Prefer not to say	1 (5.6%)
Current employment status	
Retired	8 (44.4%)
Working full-time	7 (38.9%)
Disabled	2 (11.1%)
Working part-time	1 (5.6%)
Highest level of education	
High school	3 (16.7%)
Some college	5 (27.8%)
College degree	9 (50.0%)
Master’s degree or higher	1 (5.6%)

### Finalization

The video was considered “final” after the edits based on feedback from stakeholders, as well as from the acceptability testing, were incorporated into the audio and visual elements of the video.

## Discussion

Increasing AA and Hispanic participation in clinical and genomic research is imperative to achieving health equity. Tailored messages via short videos have been demonstrated to assist in addressing the barriers and facilitators towards research participation and increase intentions to enroll in clinical trials. The culturally tailored video, informed by constructs from the HBM and EPPM, was developed through six iterative stages: 1) brainstorming/writing; 2) storyboarding; 3) animating; 4) screening/revisions; 5) acceptability testing; 6) finalization.

The use of a digital video technology to assist with the delivery of culturally tailored genomic research and clinical trial education among AA and Hispanic populations in safety-net hospitals and rural community-based settings is a novel approach. Digital video technology has been demonstrated to provide access to salient health information to underserved populations and can facilitate multicultural content integration. While the internet enables users to access health information from a variety of sources, lack of access to computers by individuals from lower socioeconomic backgrounds and the high literacy of the health information remains significant barriers. The utility of short, tailored videos ensures the delivery of standardized, accurate, relevant, and culturally and linguistically appropriate health messages. Additionally, videos allow for real-time digital updates of health information and recommendations. In comparison, Digital Versatile Disc (DVD) and printed materials such as static posters and banners are costly to replace over time, particularly if the content is to remain relevant to the target audience. Utilization of videos ensures a standard message is delivered, thus reducing the variance often associated with community-based education. Given the increasing complexity of the information regarding research participation, the use of technology will assist the delivery of a consistent message and ultimately reduce the variance.

## Conclusions

The creation of a theory-informed animated video to increase clinical trial and genomic research participation among underrepresented populations is an iterative process involving many stakeholders. The initial formal and informal feedback from the final product has been positive, and next steps are underway to test the video’s effectiveness at increasing intentions to participate in clinical trials and genomic research. Ultimately, such interventions hold promise for advancing health equity by ensuring that all individuals have equal access to health education information that may lead to more informed decisions when choosing to partake in clinical and genomic research. Future researchers who are interested in creating informative videos should consider utilizing theories of health behavior and communication to inform their script and should solicit feedback from stakeholders at each step.

### Supplementary Information

Below is the link to the electronic supplementary material.

Supplementary file1 (DOCX 9625 KB)

## Data Availability

Data will be made available upon reasonable request to the corresponding author.
