# Supplementary material for: Addressing Barriers and Facilitators to African Americans’ and Hispanics’ Participation in Clinical and Genomic Research Through a Bioethical Sensitive Video
Source: J Cancer Educ. 2024 May 2;39(4):464–70. doi: 10.1007/s13187-024-02433-w (PMC11219413; doi:10.1007/s13187-024-02433-w)
Supplement: Supplementary file 1 — Supplementary file1 (DOCX 9625 KB) [file 13187_2024_2433_MOESM1_ESM.docx]

| **Supplement 1.** Screenshots and accompanying text from video | |
| --- | --- |
| 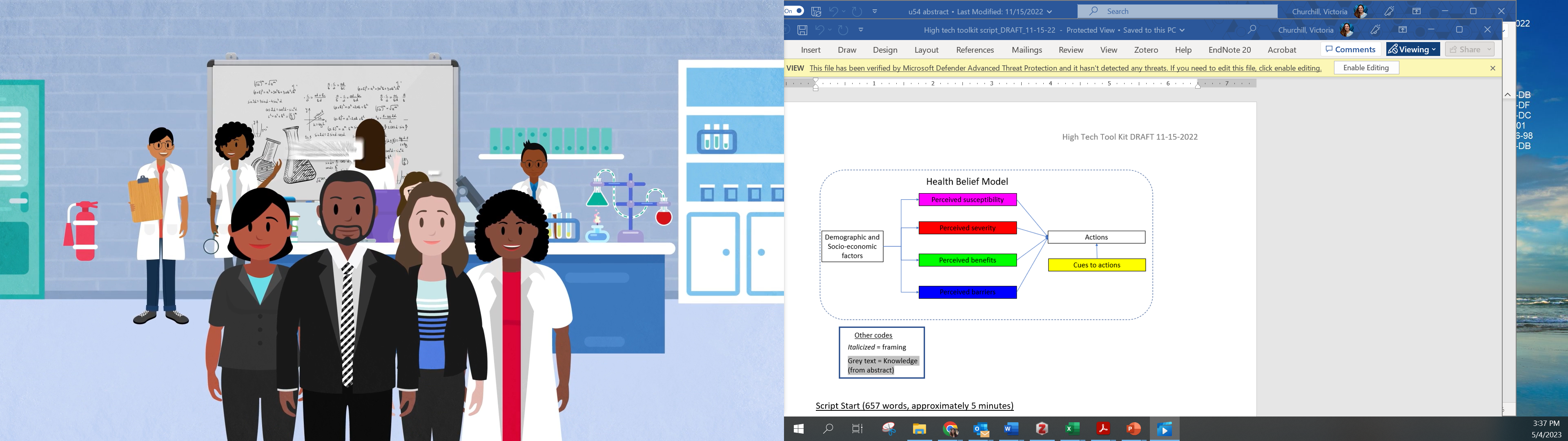   1. “Doctors and scientists are working on advances that have the potential to improve our communities’ health and well-being.” | 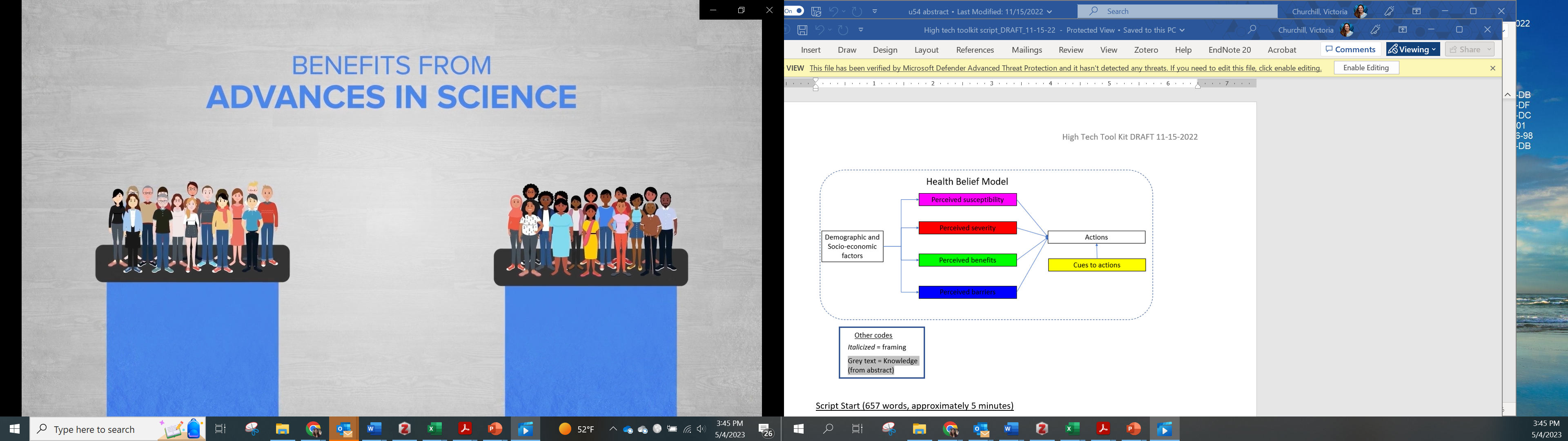   1. “This can lead to and widen health disparities…” |
| 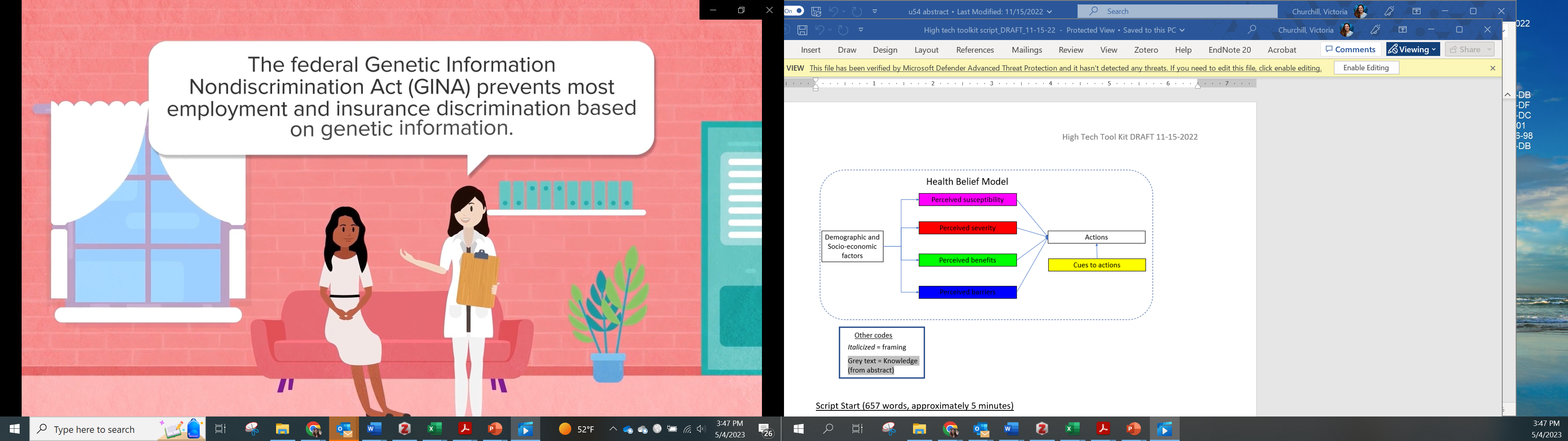   1. “For example, the federal Genetic Information Nondiscrimination Act prevents most employment and insurance discrimination based on genetic information.” | 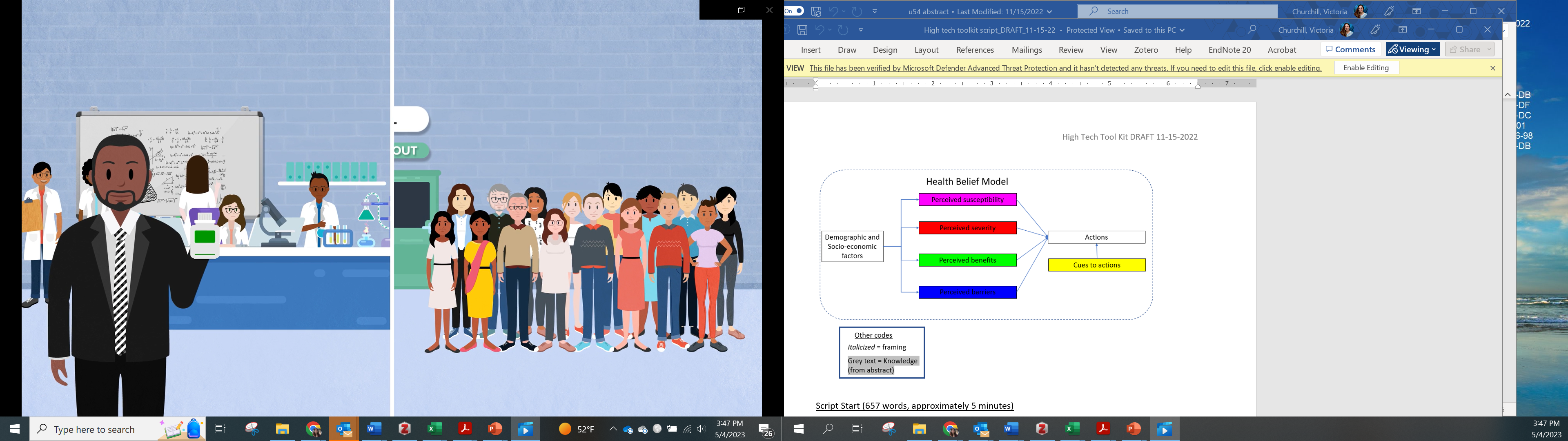  d. “Many people find comfort knowing that by donating samples, they will help researchers make discoveries that can advance medicine and improve lives of people in their community, now and in the future.” |
